# MXene-based composite photocatalysts for efficient degradation of antibiotics in wastewater

**DOI:** 10.1038/s41598-024-83333-3

**Published:** 2024-12-28

**Authors:** Masoud Akbari, Jamal Rasouli, Kamal Rasouli, Samaneh Ghaedi, Milad Mohammadi, Hamid Rajabi, Samad Sabbaghi

**Affiliations:** 1 Department of Nano-Chemical Engineering, Faculty of Advanced Technologies, Shiraz University, Shiraz, Iran; 2 Nanotechnology Research Institute, Shiraz University, Shiraz, Iran; 3 Department of Chemical Engineering, School of Chemical and Petroleum Engineering, Shiraz University, Shiraz, Iran; 4 Department of Civil Engineering and Management, the University of Manchester, Manchester, M13 9PL UK; 5 Department of Civil and Environmental Engineering, School of Engineering, University of Liverpool, Liverpool, L69 3GH UK; 6 School of Engineering, Harrison Hughes Building, University of Liverpool, Liverpool, L69 3GH UK; 7 Faculty of Advanced Technologies, Shiraz University, Shiraz, Iran

**Keywords:** MXene, Photocatalyst, Antibiotics, Tetracycline, Wastewater treatment, Chemical engineering, Environmental chemistry

## Abstract

MXene-based (nano)materials have recently emerged as promising solutions for antibiotic photodegradation from aquatic environments, yet they are limited by scalability, stability, and selectivity challenges in practical settings. We formulated Fe_2_O_3_-SiO_2_/MXene ternary nano-photocomposites via coupled wet impregnation and sonochemistry approach for optimised tetracycline (TC) removal (the second most used antibiotic worldwide) from water using response surface methodology-central composite design (RSM-CCD). The photocatalysts containing various loading of Fe_2_O_3_/SiO_2_ (5–45 wt%) on the MXene with a range of calcination temperatures (300–600 °C) via RSM optimisation were synthesised, characterised regarding crystallinity properties, surface morphology, binding energy, and light absorption capability, and analysed for TC degradation efficiency. The 25FeS/MX-450 composite among all samples demonstrated a superior efficiency in TC photocatalytic removal (98%) under optimised conditions (TC degradation: 39.75 mg/L, time: 68.28 min, pH: 5.57, catalyst dosage: 0.75 g/L). The developed surface area, with a reduced band gap due to FeS nanoparticles incorporation with improved light absorption within the visible spectrum, played a crucial role in the 25FeS/MX-450 heterostructure matrix, enhancing photogenerated carriers’ separation and transportation capabilities. The tetracycline photoreduction mechanism involved electron transfer from FeS to the surface of MXene, engaging with O_2_ to produce •O_2_−, attributed to the high electron mobility of MXene. Our findings for such nano-photocomposites materials can underscore the considerable potential of MXene-based nanomaterials for pharmaceutical removal from waterways.

**Supplementary Information:**

The online version contains supplementary material available at 10.1038/s41598-024-83333-3.

## Introduction

Rapid technological progress across industrial and medical sectors has significantly contributed to the increasing discharge of refractory antibiotics into water resources. Antibiotics are widely used in human medicine, agriculture, and animal husbandry, which are crucial in treating infections in humans and animals ^2^. However, due to the incomplete metabolic breakdown, residual antibiotics can remain in aquatic environments. This can lead to antimicrobial resistance, reduce antibiotic efficacy, and threaten the ecosystem^5^. Tetracycline (TC), the second most widely used antibiotic, is frequently released into water sources due to human activities^16,29^.

Conventional methods for antibiotic removal, such as adsorption, evaporation, electrochemical, and biological treatments, often have limitations, including poor resource recovery, incomplete degradation to safe levels^39,41,^^44^, and formation of potentially toxic by-products, like chloro-phenyl-isoxazole in electrochemical treatments^43^. Recent studies, including approaches using nanohybrids and photocatalysts, offer alternative methods with improved degradation efficiency and environmental safety^27,40^. Photocatalytic degradation^3,46^, one of the advanced oxidation processes (AOPs), has emerged as a promising method for completely degrading antibiotics into CO_2_ and H_2_O. In this method, light is used as an energy source and non-polluting metals are employed as catalysts to effectively mineralise various pollutants into more minor and non-toxic compounds. Semiconductors, such as Zinc (Zn), iron (Fe), Silicon (Si), Titanium (Ti), and Bismuth (Bi), are commonly utilised as photocatalysts due to their high efficiency, simple production, and suitability for heterogeneous processes^19,20^. Fe_2_O_3_ has been identified as a distinctive non-toxic photocatalytic semiconductor characterised by its narrow band gap. Furthermore, Fe_2_O_3_ benefits from various properties such as high chemical stability, remarkable electrical conductivity, abundant availability within the earth’s crust, and cost-effectiveness compared to other semiconductor photocatalysts. It should be highlighted that surface modification with mesoporous SiO_2_ is a viable option compared to other coating materials, which can play a versatile carrier option due to its capability to enhance biocompatibility, reduce the level of agglomeration, and increase the stability of nanoparticles^23–26,38,46^.

Heterogeneous photocatalysis has become a widely adopted strategy for pollutant removal, utilising materials such as TiO_2_, ZnO, and Fe_2_O_3_ due to their stability and effectiveness. However, these materials often face limitations due to rapid electron-hole recombination, which reduces their photocatalytic efficiency and restricts their practical applications^7^. To overcome these limitations, recent approaches incorporate carbon-based and hybrid composites, which enhance charge separation, extend light absorption, and improve overall catalytic performance^31^. This study builds on these advancements by synthesising a Fe_2_O_3_-SiO_2_/MXene nanocomposite tailored for efficient tetracycline degradation under visible light, demonstrating the potential for enhanced environmental applications^30,44^.

Researchers have recently focused on enhancing the performance of photocatalysts by utilising cost-effective metals and two-dimensional nanomaterials such as graphene oxides, carbon nanotubes, metal-organic frameworks, and MXenes (a new class of 2D metal carbides/nitrides) to reduce the production cost and increase the stability. Among these materials, MXene-based nanomaterials have attracted significant attention due to their unique properties, including strength, large specific surface area, flexibility, biocompatibility, high melting point, large interlayer spacing, hydrophilicity, and ability to form composites^12,23–26,52^. Due to their high electrochemical activity, porous structure, and environmental adaptability, MXenes have been used as co-catalysts in photocatalysis, effectively reducing the recombination of electron-hole pairs and improving the efficiency of photocatalytic processes. Nonetheless, some studies have investigated the degradation of tetracycline using MXenes in aquatic environments^4,12,21,34,48^.

This study examines the development of a novel Fe_2_O_3_/SiO_2_/MXene nano-photocomposite designed for superior photocatalytic degradation of TC from wastewater. We have employed a combined sonochemical and wet impregnation method for a straightforward and potentially scalable synthesis strategy. This represents the first application of the response surface RSM-CCD method to optimise the loading of Fe_2_O_3_/SiO_2_ on the MXene support, offering a data-driven approach towards achieving optimal photocatalytic activity for antibiotic removal. The combination of Fe_2_O_3_, SiO_2_, and MXene in the composite leverages the photocatalytic benefits of each component: Fe_2_O_3_ for visible light absorption and ROS generation, SiO₂ for structural support and stability, and MXene for enhanced electron mobility and charge separation. These features work synergistically to improve the composite’s efficiency in degrading tetracycline in wastewater.

A wide range of characterisation techniques, including XRD, BET, SEM equipped with EDS mapping, and DRS, have been conducted to comprehensively understand the synthesised materials and their photocatalytic performance. The results of the characterisation techniques, along with RSM-CCD optimisation, provide a robust foundation for achieving efficient TC photodegradation. Furthermore, we have investigated the effects of operating conditions, kinetics, TC degradation mechanisms, and regeneration ability using Fe₂O₃/SiO₂/MXene photocatalyst, which shows the potential of this novel photocatalyst in real wastewater.

This study presents a novel Fe_2_O_3_-SiO_2_/MXene composite photocatalyst, uniquely engineered and optimised for efficient degradation of tetracycline, a common antibiotic contaminant in wastewater. We have developed a composite with synergistic properties by combining MXene, which offers a high surface area, conductivity, and rapid electron transport, with Fe_2_O_3_-SiO_2_, which enhances visible light absorption and reactive oxygen species (ROS) generation. This innovative approach leverages the strengths of each component to create a potent photocatalyst, marking a significant advancement in antibiotic removal from water. To our knowledge, this particular combination and optimisation have not been previously reported, but a new avenue in MXene-based photocatalyst research for environmental remediation has been established.

## Materials and methods

### Materials

SiO_2_ nanoparticles were obtained from US Research Nanomaterials Inc., USA. Fe (NO_3_)_3_.9H_2_O (99% purity) was acquired from Sigma-Aldrich, USA, while absolute ethanol (99.9% purity) was also obtained from Sigma-Aldrich, USA. The source for TC utilised in this study is the Iranian Farabi Pharma Company, Iran. AgNO_3_ (99% purity), EDTA and benzoquinone were procured from Merck, Germany. Ti_3_AlC_2_ (> 99% purity) was sourced from American Elements, USA, whereas HF (40% purity), HCl (37% purity), and NaOH were obtained from Sigma-Aldrich, USA. It is important to note that all chemicals were used as received without further purification.

### SiO2 synthesis

In a laboratory setting, a mixture was prepared by combining 18.8 mL of deionised water (DI), 25 mL of pure ethanol, and 19 mL of ammonia water in a beaker^9^). Subsequently, the solution was agitated for 15 min at an average speed of 800 revolutions per minute (rpm). Another step in the process involves the gradual addition of 1.8 mL of tetraethoxysilane (TEOS) to an Erlenmeyer flask, followed by continuous stirring of the mixture for an additional 30 min. The reaction proceeded for an extra two hours. Upon completion, the resultant product underwent centrifugation followed by multiple washing cycles with DI water and ethanol until complete purification was achieved. Finally, the material was lyophilised, resulting in a powdered form consisting of dried SiO_2_ nanoparticles.

### MXene synthesis

Ti_3_C_2_Tx MXene was synthesised using a widely recognised method as reported in the literature^22,35^. The synthesis process involved etching the Ti_3_AlC_2_ MAX phase precursor with an HF solution. One gram of the Ti_3_AlC_2_ MAX phase was gradually added to a beaker containing the HF solution, allowing the mixture to bubble for 5 to 10 min. The resulting solution was then stirred at room temperature for 8 h. The resulting powder was subsequently washed multiple times with DI water and centrifuged until a neutral pH was achieved. Finally, the sediment was dried under nitrogen (N_2_) flow overnight.

### FeS/MXene synthesis

**Fig. 1:**
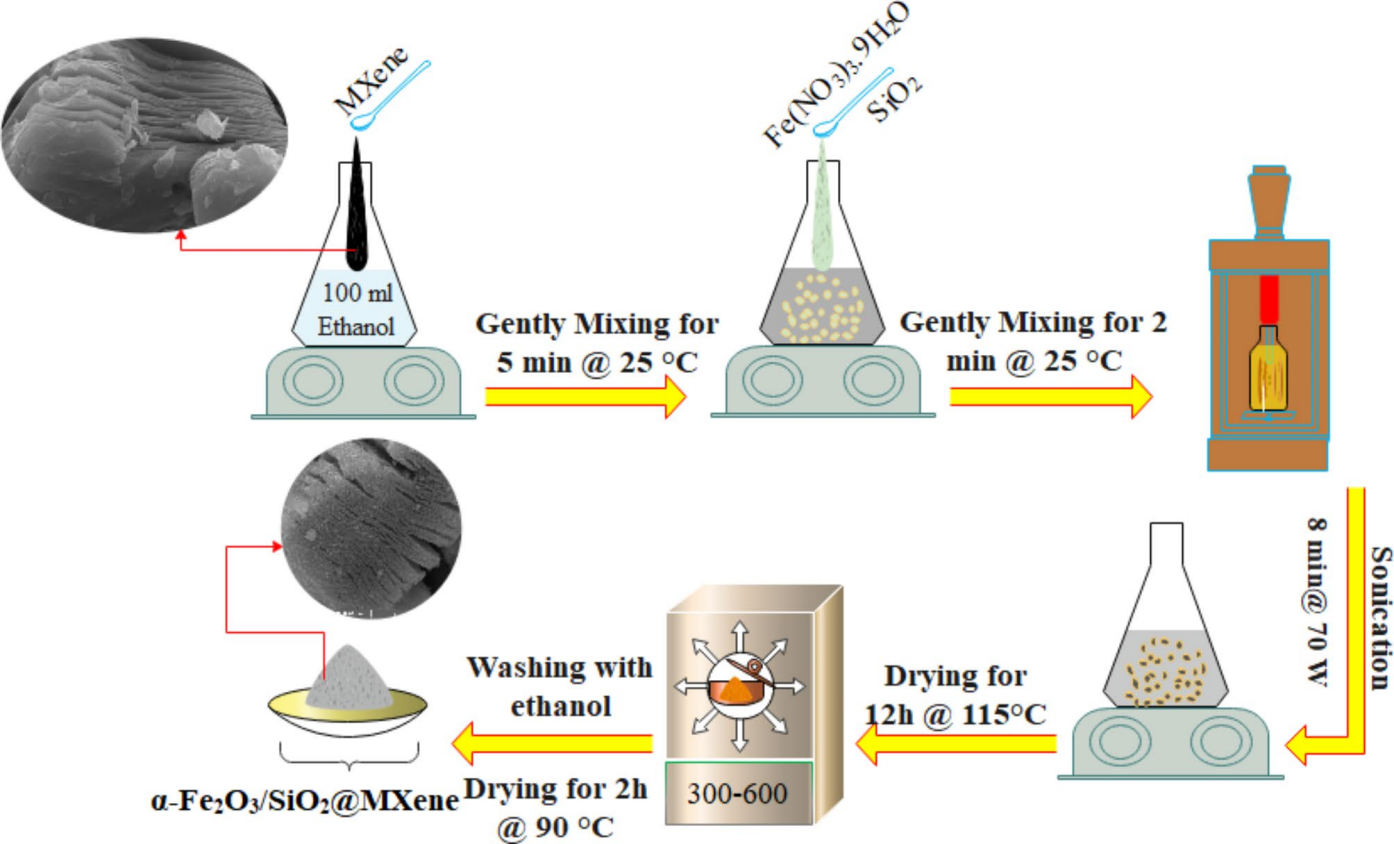
Synthesis procedure of FeS/MXene photocatalyst.

The FeS nanocomposite was produced using a synthesis method that involved ultrasound assistance and the wet impregnation technique. This method involved a commercially available SiO_2_ source with dissolved Fe (NO_3_)_3_·9H_2_O in ethanol to create α-Fe_2_O_3_ (Fig. 1). Different weight percentages of α-Fe_2_O_3_ (1, 3, 5, and 7 wt%) were obtained, and the resulting nanocomposites were subjected to varying calcination temperatures (300 ◦C). In the synthesis of FeS/MXene, a solution was created by stirring 1 g of synthesised MXene in 100 ml of ethanol for 5 min using a magnetic stirrer. Subsequently, the appropriate amount of FeS was added to the solution based on stoichiometric measurements. The resulting mixture was then stirred for 1 h at ambient temperature and atmospheric pressure, followed by sonication at 70 W for 8 min. Following these steps, the ethanol was evaporated overnight at 115 °C. At the final phase of the process, mesoporous nanocomposites of FeS/MXene with varying weight percentages of 5, 15, 25, 35, and 45 (e.g., Fe_2_O_3_/SiO_2_/MXene: 300/75/125 mg) were prepared by annealing the FeS/MXene powder at temperatures ranging from 300 to 600 °C for 3 h. Prior to calcination, the powder was washed with ethanol and dried at 90 °C for 2 h. Subsequently, the composites were evaluated through a range of characterisation techniques for their photocatalytic properties.

### RSM optimised nanocomposite synthesis

To optimise the synthesis of the FeS/MXene photocatalyst, an initial exploratory phase was undertaken to identify the critical parameters influencing its formation. A central composite design (CCD) was then employed to systematically assess the impact of two key variables: calcination temperature and FeS loading on the MXene support. CCD was chosen as the preferred response surface methodology (RSM) due to its established effectiveness in constructing second-order models. This approach utilised five levels (−2, −1, 0, 1, 2) for each chosen variable, as detailed in Table S1. Notably, all other synthesis parameters, including the power level known to affect nanocomposite morphology, remained constant during the optimisation process. Table 1 summarises the specific preparation conditions used for each catalyst sample. Subsequently, photocatalytic degradation tests employing a target contaminant (TC) were conducted to identify the combination of parameters yielding the most efficient photocatalyst. It’s crucial to note that the FeS proportions were meticulously chosen to ensure the formation of the desired α-phase iron oxide.

### Characterisation techniques

X-ray diffraction (XRD) analysis was conducted using the X’Pert Pro instrument from Panalytical (Netherlands) to investigate the crystal structure phase composition of the synthesised and sintered nanocomposites. Surface characteristics such as specific surface area, pore volume, and average pore diameter of the catalyst support and fully prepared catalysts were determined through Brunauer-Emmett-Teller (BET) surface analysis using Japan’s BELSORP Mini II instrument. The morphology of the synthesised samples was evaluated using transmission electron microscopy (TEM) (EM208S, Philips, Netherlands) and scanning electron microscopy (SEM) (MIRA3, TESCAN, Czech Republic). Energy dispersive spectrometry (EDS-map) analysis, performed using the VEGA3 instrument from TESCAN in the Czech Republic, was employed to quantify the actual loaded quantity of different elements on the catalyst support. The optical properties of the synthesised nanocomposites were evaluated using a UV-Vis diffuse reflectance spectrophotometer (V-730, Jasco, USA). Furthermore, the band gap energy, which represents the energy difference between the valence and conduction bands and plays a critical role in determining the photocatalytic activity, was calculated using Eq. (1):

1 $$\:O\left(\alpha\:hv\right)={B(hv-{E}_{g})}^{n}$$

Where α, h, ν, and E_g_ correspond to the absorption coefficient, Planck’s constant, light frequency, and bandgap energy, respectively. The absorption coefficient α is determined as 2.303 A/d, where A denotes the absorbance wavelength and d is taken as 1 for a standard quartz cell. The variable hν is obtained using the equation hν = hc/λ, where c denotes the speed of light (3 × 10^8 m/s), h is Planck’s constant (6.6261 × 10^−34^ J.s), and λ is the wavelength (nm). The constant B and the variable n are also utilised, where n representing the type of transition (0.5 or 2 for direct and indirect semiconductors) within the linear Tauc region of the optical absorption peaks. Additionally, the coefficient hν can be computed using the equation hν = 1240/λ (nm).

Additionally, a UV-Vis spectrophotometer was utilised to measure the absorbance and concentrations of TC using the following equation (Eq. 2):

2 $$\:TD\left(\%\right)=(1-\frac{{C}_{t}}{{C}_{0}})\times\:100$$

where TD represents the degradation efficiency of TC and C_0_ and C_t_ are the initial and t-time concentrations of TC, respectively.

### Photodegradation experiments

The adsorption capacities, isotherms, and kinetics of materials for TC antibiotics were evaluated by varying dosages (0.1–1.3 g/L), pH levels (2–10), Time (20–140 min), and pollutant concentrations (10–70 mg/L) in a 20 mL aqueous solution of TC. The concentration of TC in the solution was determined using a UV–vis spectrophotometer after separation of the composite. Additionally, the photocatalytic performance of each sample was evaluated by monitoring the degradation of TC solution. During this evaluation, 20 mL of TC solution with concentrations ranging from 10 to 70 mg/L and a specified amount of the synthesised photocatalyst were mixed in a 30 mL beaker. The mixture was then exposed to visible light emitted by a 500 W halogen source (> 400 nm) for a specific duration at a defined pH level. The initial pH values of the solutions were adjusted using diluted solutions of HCl and NaOH^17,37^. To achieve equilibrium between the TC solution and the photocatalyst, the solution underwent magnetic agitation in a light-restricted environment for 30 min before exposure to visible light. The adsorption rate of TC demonstrated a stable trend, maintaining a consistent level of 24% following an initial 30-min period of darkness. Subsequently, 3 mL of the suspension was extracted at different time intervals, including both the period of darkness and the duration of irradiation, to evaluate the photocatalytic efficacy of the synthesised photocatalysts. Before evaluating the concentrations of TC, the samples were subjected to centrifugation at 7500 rpm for 10 min to ensure complete separation of the photocatalyst.

## Result and discussion

### Composites activity

**Fig. 2:**
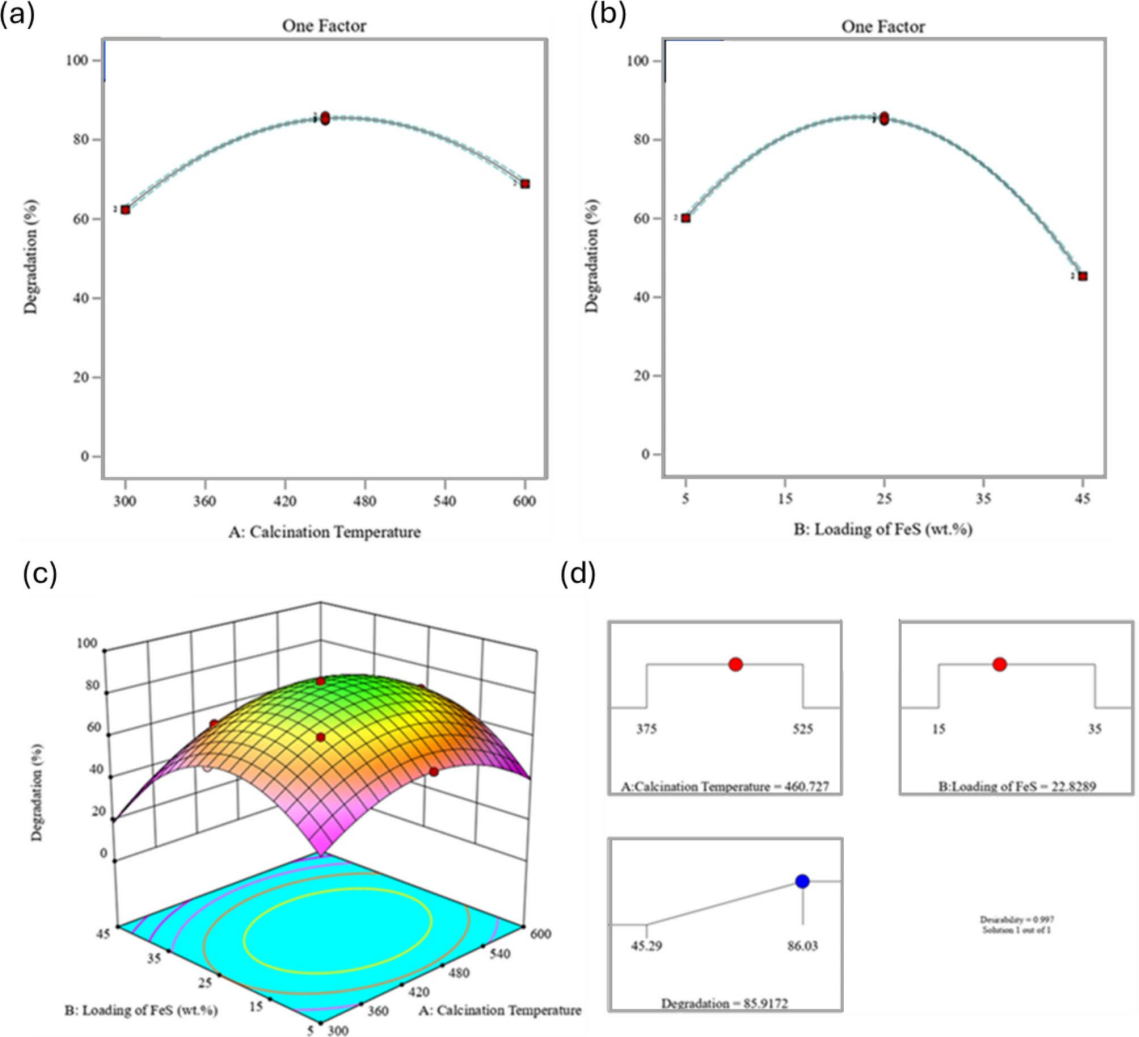
(**a**) The effect of FeS loading and (**b**) calcination temperature of synthesised photocatalysts on the removal of TC, (**c**) interaction between calcination temperature and FeS loading, and (**d**) Optimal quantities of parameters in the synthesis of the nanocomposite (further information in the supplementary materials).

The study evaluated the effectiveness of synthesised nanocomposites for the photocatalytic degradation of TC under visible light irradiation. A series of 13 different nanocomposites were synthesised and their photocatalytic activity was assessed using a halogen lamp. The optimal photocatalyst was identified among these nanocomposites, and the experimental details are provided in Table 1. The photocatalytic reaction solution was prepared by dispersing 0.8 g/L of the photocatalyst in 20 mL of a 20 mg/L TC solution. The irradiation time was 80 min, and the pH value was maintained at 5. Notably, using MXene resulted in an adsorption efficiency of 31.3% under dark conditions, while a photocatalytic degradation efficiency of 54.26% was achieved. The results, as depicted in Fig. 2a-c, indicated that the optimal loading of FeS and calcination temperature for the synthesis of the FeS/MXene nanostructure were 25 wt% and 450 °C, respectively. Furthermore, both Table 1; Fig. 2c illustrated that centre high efficiency of TC degradation (86.03%) was attained at centre point. In conclusion, the successful synthesis of the nanocomposites improved their photocatalytic capability in the visible region, surpassing the performance of pure MXene (54.26%) in terms of photocatalytic degradation. The influence of FeS content and calcination temperature on the photocatalytic efficiency of the synthesised materials was corroborated by the data presented in Fig. 2. Table 1 revealed a significant enhancement in TC degradation yield, increasing from 60.14 to 86.03%, as the FeS loading was progressively raised from 5 wt% to 25 wt%. However, a further increase in loading to 45 wt% resulted in a decline in efficiency to 45.29%. Conversely, Fig. 2c depicts a moderate improvement in TC degradation (from 62.29 to 86.03%) with increasing calcination temperature (300 °C to 450 °C) for a fixed FeS loading (25 wt%). However, a significant decrease to 68.92% was observed when the temperature was further elevated to 600 °C. An optimal combination of parameters for the synthesis of the 25FeS/MX-450 nanocomposite was identified through data analysis (Fig. 2d) to be 25 wt% FeS loading and a calcination temperature of 450 °C. Our investigation revealed that the amount of FeS incorporated (FeS loading) significantly impacted the photocatalytic performance more than the calcination temperature. The FeS loading range (5–45 wt%) and calcination temperature range (300–600 °C) were selected after thorough experimentation, referencing existing studies, and validation with RSM-CCD. This approach aimed to optimize factors like surface area, active site availability, and crystallinity, all essential for efficient tetracycline degradation. Lower FeS loadings (under 5 wt%) limited active sites, while higher loadings (over 45 wt%) caused agglomeration, reducing the effective surface area. Similarly, calcination below 300 °C did not provide enough crystallinity, and temperatures above 600 °C led to structural changes. This carefully balanced combination supports both effective and stable photocatalytic performance.

**Table 1:** List of nanocomposites synthesised and their photocatalytic activity in TC degradation.

Catalyst	Calcination Temperature (300–600 °C)	FeS Loading (5–45 wt%)	Degradation (%)
25 FeS/MX-600	600	25	68.92
25 FeS/MX-450	450	25	84.77
25 FeS/MX-300	300	25	62.29
25 FeS/MX-450	450	25	85.15
45 FeS/MX-450	450	45	45.29
5 FeS/MX-450	450	5	60.14
25 FeS/MX-450	450	25	86
15 FeS/MX-375	375	15	75.36
35 FeS/MX-375	375	35	66.18
25 FeS/MX-450	450	25	86.03
15 FeS/MX-525	525	15	76.56
35 FeS/MX-525	525	35	71.14
25 FeS/MX-450	450	25	85

### Characterisation of photocatalysts

#### XRD

**Fig. 3:**
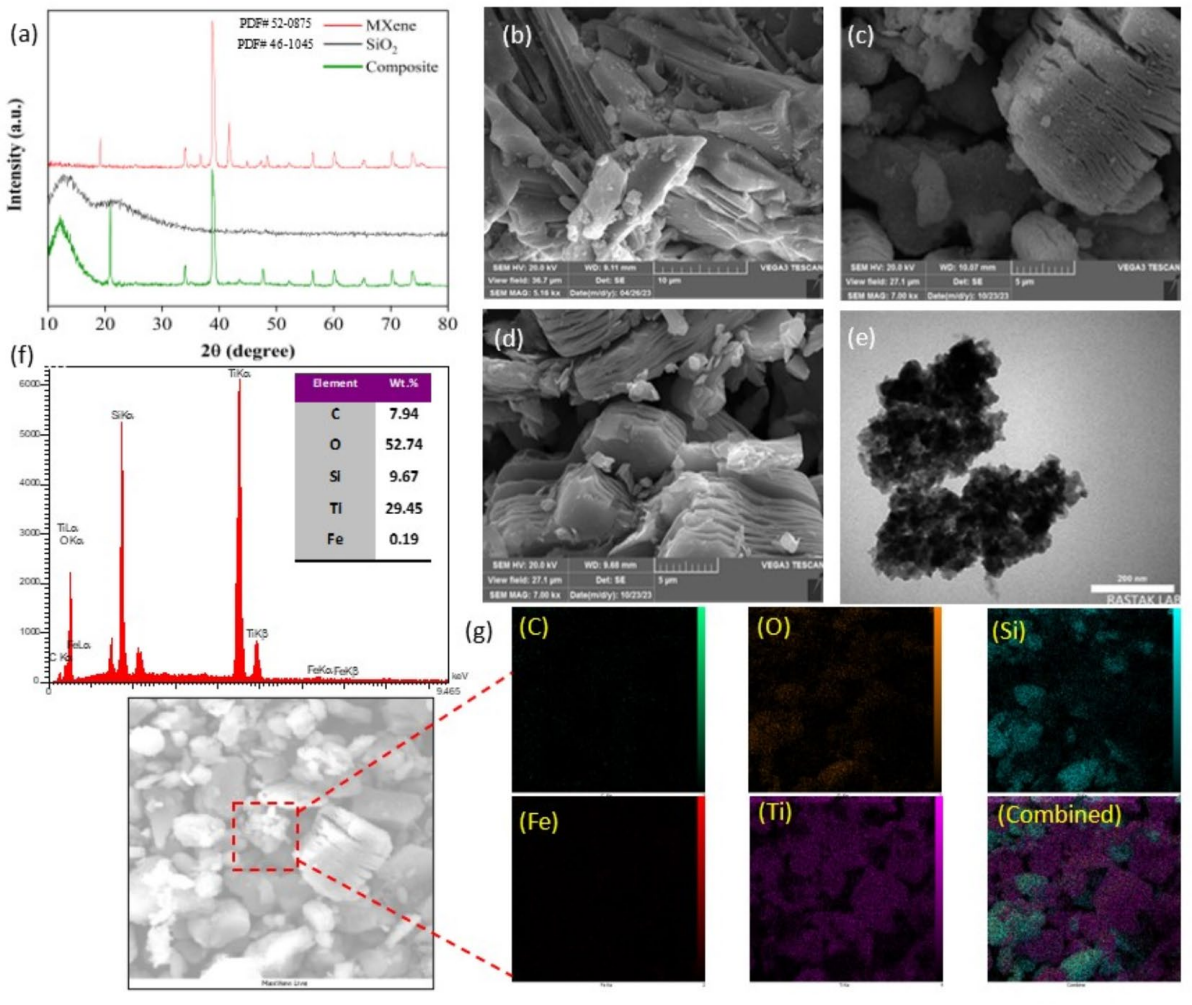
(**a**) XRD pattern of materials, SEM image of (**b**) Ti_3_AlC_2_ before etching (**c**) MXene, and (**d**) 25FeS/MX-450, (**e**) TEM image of 25FeS/MX-450, (**f**) result of EDX analysis of 25FeS/MX-450, (**g**) Elemental mapping of C, O, Si, Ti, Fe and combine of the 25FeS/MXene- 450, nanocomposite.

The chemical composition and phase structure of the synthesised materials were analysed using X-ray diffraction (XRD) techniques. The XRD analysis confirmed the successful synthesis of the 25FeS/MX-450 composite, as demonstrated in Fig. 3a. The crystal structure and purity of the synthesised mesoporous composite in the MXene phase were assessed through XRD examination. In Fig. 3a, distinct peaks were observed at 2θ angles of 18.90°, 34.12°, 36.22°, 38.8°, 41.81°, 48.43°, and 60.25°, corresponding to the (0 0 4), (1 0 1), (1 0 3), (1 0 4), (1 0 5), (1 0 7), and (1 0 9) planes of the MXene phase. The absence of a sharp peak in Fig. 3a is attributed to the amorphous nature of the SiO_2_ particles, which instead exhibited a broad peak at a wide diffraction angle of 20° to 30°. Furthermore, the XRD pattern of the as-prepared 25FeS/MX-450 nanocomposite revealed the presence of peaks corresponding to Fe_2_O_3_ and SiO_2_ within the composite.

#### SEM, EDX, and TEM analysis

SEM analysis was employed to elucidate the morphological transformation induced by the selective etching process. Figure 3b reveals the initial layered and densely packed structure of Ti_3_AlC_2_, consisting of titanium, aluminium, and carbide elements. The etching process selectively weakens the Ti-Al bonds, facilitating the removal of aluminium and the formation of interlayer spaces within the resulting Ti_3_C_2_ (Fig. 3c). This accordion-like morphology of Ti_3_C_2_ clearly contrasts with the compact, layered structure of the precursor (Ti_3_AlC_2_), visually confirming the effectiveness of the etching process. Moreover, the integration of FeS onto the MXene is clearly observed, as depicted in Fig. 3d. Additionally, EDS analysis of the 25FeS/MX-450 photocatalyst (Fig. 3f) provides information on the elemental distribution. The analysis confirms the presence of iron (1.19 wt%) and a significant percentage of oxygen in the 25FeS/MX-450 (48.74 wt%). These results confirm the successful incorporation of 25 wt% FeS onto the MXene structure. Moreover, the complete removal of aluminium is evident from the absence of any corresponding peak in the EDS spectra of the composite material. The morphology and the interaction between components in the composite photocatalyst were investigated by TEM, as shown in Fig. 3e. As expected, Ti_3_C_2_ exhibits a plate-like morphology, while the α-Fe_2_O_3_ nanoparticles appear spherical and well-distributed on its surface. The absence of distinct layering in the Ti_3_C_2_ structure suggests limited penetration of α-Fe_2_O_3_/SiO_2_ nanoparticles within its internal structure. Furthermore, the elemental mapping results of the 25FeS/MX-450 composite reveal effective dispersion of Fe and Si elements on the MXene support, indicating successful impregnation of FeS, as illustrated in Fig. 3g. This observation aligns with the anticipated behaviour, where the nanoparticles primarily reside on the surface or within easily accessible pores of the Ti_3_C_2_ support.

#### BET and UV–Vis spectroscopy analysis

**Fig. 4:**
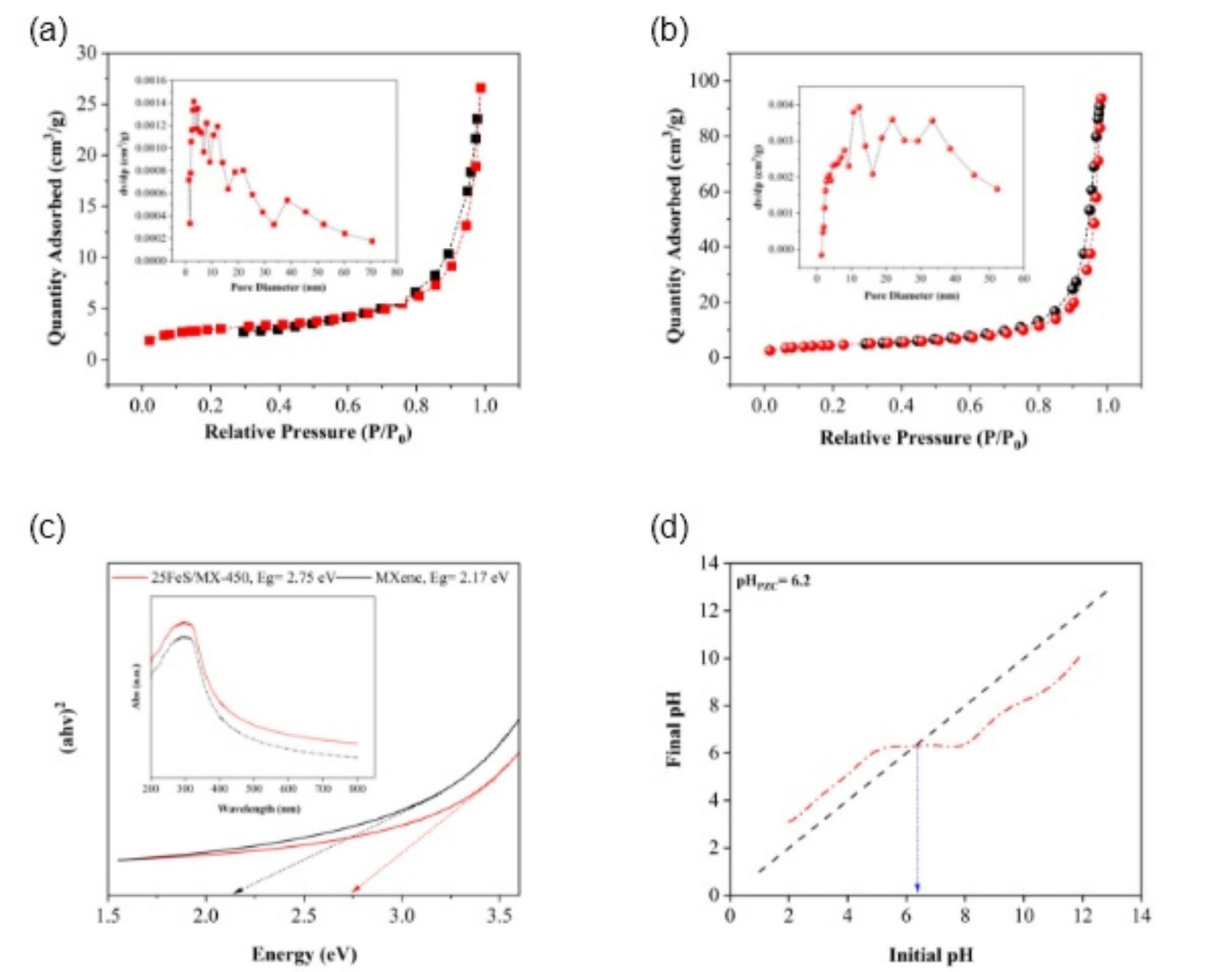
Ads/Desorption curves of (**a**) MXene and (**b**) 25FeS/MX-450 photocatalyst, (**c**) energy band gap for MXene and 5FeS/MX-450, (**d**) Plot of pHpzc of 5FeS/MX-450.

As shown in Table 2, the MXene material exhibits a pore diameter of 8.34 nm and a pore volume of 0.07 cm^3^.g^−1^. Following impregnation with FeS, the composite’s pore diameter and pore volume increased to 0.14 cm^3^.g^−1^ and 21.58 nm, respectively. Moreover, the BET surface area of the MXene material increased from 22.05 m^2^. g^−1^ to 26.7 m^2^. g^−1^, which can be contributed to the improved photocatalytic performance of the composite by increasing the available surface area for the adsorption of pollutant molecules. Figure 4a-b depicts the N_2_ adsorption-desorption isotherms of MXene and the 25FeS/MX-450 nanocomposite. The isotherms exhibit type IV characteristics for both materials, indicating a mesoporous structure according to the IUPAC classification.

Hematite (α-Fe_2_O_3_), the most thermodynamically stable iron oxide under ambient conditions, exhibits a narrow band gap (Eg ≈ 2.2 eV), enabling it to absorb visible light. This property makes it a promising candidate for photocatalysis applications under visible light irradiation. However, the recombination of photogenerated electron-hole pairs remains a significant challenge. To address this limitation, the study explores the use of MXene as a base of catalyst and Fe_2_O_3_ as a co-catalyst. The strong interaction between MXene layers and α-Fe_2_O_3_/SiO_2_ nanoparticles is expected to facilitate the separation of electron-hole pairs and improve charge transport, thereby enhancing the overall photocatalytic performance. An investigation of the optical properties of the employed materials, including MXene, SiO_2_, and 25FeS/MX-450, provides valuable insights into their potential applications. As shown in Fig. 4c, the measured band gap values are approximately 2.17 eV for MXene and 2.75 eV for 25FeS/MX-450. The narrower band gap of 25FeS/MX-450 suggests a greater propensity for visible light absorption, potentially due to the presence of Fe_2_O_3_ and MXene nanomaterials with inherently lower band gaps.

SPSS analysis revealed that surface area and photocarrier separation both had strong positive correlations with degradation efficiency, with correlation coefficients of *r* = 0.88 and *r* = 0.91, respectively (*p* < 0.01). These high correlation values suggest that an increased surface area significantly enhances the availability of active sites for pollutant interaction, thereby promoting higher photocatalytic efficiency. Similarly, effective photocarrier separation reduces electron-hole recombination, which is crucial for sustaining reactive oxygen species (ROS) production, directly impacting the degradation process. Conversely, the band gap demonstrated a moderate negative correlation with degradation efficiency (*r* = −0.62, *p* < 0.05), indicating that a smaller band gap, which allows for greater absorption of visible light, contributes positively to photocatalytic activity. This relationship underscores the importance of designing materials with narrower band gaps to harness a broader spectrum of light, thereby boosting the overall photocatalytic efficiency. These statistical results collectively highlight the critical roles of surface area, photocarrier separation, and band gap in optimizing the composite’s performance, guiding future design considerations for high-efficiency photocatalysts.

**Table 2:** Surface properties of MXene and synthesised composites.

Sample	S_BET_ (m^2^.g^−1^)	Pore volume (cm^3^.g^−1)^	Pore diameter (nm)
MXene	22.05	0.07	8.34
25FeS/MX-450	26.7	0.14	21.58

#### The measurement of pHpzc

The pH drift approach was employed to determine the pHpzc (point of zero charge pH) for the 25FeS/MX-450 composite. The pHpzc represents the pH at which the catalyst’s surface becomes neutrally charged, a critical parameter in assessing its behaviour. Meaning that the composite’s surface is positively and negatively charged before and after the pHpzc, respectively. The determined pHpzc for the 25FeS/MX-450 composite is 6.2, indicating that the composite is negatively charged at pH levels above this value and positively charged at pH levels below it. In this study, a 25 mL volume of 0.1 M NaCl solution was initially prepared within a pH range of 2 to 12, with pH adjustments carried out using 1 M NaOH or HCl solutions. Subsequently, 0.05 g of the composite was added to each solution. After 48 h of agitation, the pH values of the solutions were measured and plotted against the initial pH values. The point of intersection of the final pH versus the initial pH curve (refer to Fig. 4d) was used to determine the pHpzc of the 25FeS/MX-450 composite.

### Photocatalytic degradation results

#### ANOVA

RSM modelling was employed to quantify the influence of various factors on TC degradation. The experimental data served as the foundation for the model, encompassing diverse levels of TC removal at different combinations of four independent variables. The model’s adequacy was rigorously evaluated using statistical measures, including adjusted R-squared, p-value, and lack of fit index, as outlined in the reference^42^. Table S2 details the experimental design employed for TC degradation and the corresponding results obtained from Design-Expert software.

Interactions between variables were deliberately excluded to ensure model parsimony and avoid overfitting. The statistical significance of the proposed quadratic model was confirmed through ANOVA analysis, with most F-values being large and p-values consistently below 0.0001. Furthermore, a robust association between observed and predicted responses for TC degradation was evident from the highly significant regression model (p-value < 0.00001), high coefficient of determination (R² = 0.9886), and well-adjusted R² value (0.9780)^18,59^. Additionally, the relatively low coefficient of variation (CV = 3.31%) indicated a balance between data spread and accuracy around the mean, further supported by the acceptable precision value of 31.39 compared to a benchmark of 4.

According to the variance analysis (ANOVA), all parameters had a statistically significant impact on the efficiency of photodegradation. The F-values identified the significant sequence of independent variables, with catalyst dosage and pH emerging as the most crucial operational variables for degrading TC. Fig. S1a-b illustrate the fitting of the anticipated and observed TC degradation rates and the normal probability plot of the residuals. The observed outcomes did not demonstrate a statistically significant variance from their anticipated counterparts. The model’s validity was further verified by residual analysis, which revealed a normal distribution of errors, signifying a lack of systematic bias. Table 3 provides the actual regression model equations. These equations represent second-order polynomial functions employed to predict the response (TC degradation efficiency) based on the interplay of the investigated independent variables. Equation 3 provides the optimised formula for the dependent variable (response):

3 $$\begin{array}{l}\text{Y}=\times\:{\text{X}}_{1}+3.26\times\:{\text{X}}_{2}-2.48\times\:{\text{X}}_{3}-2.71\times\:{\text{X}}_{4}+0.35\\ \quad\quad \times\:{\text{X}}_{1}{\text{X}}_{2}-4.21\times\:{\text{X}}_{1}{\text{X}}_{3}+1.15\times\:{\text{X}}_{1}{\text{X}}_{4}-2.64\times\:{\text{X}}_{2}{\text{X}}_{3}-8.97\\ \quad\quad \times\:{\text{X}}_{2}{\text{X}}_{4}+7.29\times\:{\text{X}}_{3}{\text{X}}_{4}-8.33\times\:{\text{X}}_{1}^{2}-4.93\times\:{\text{X}}_{2}^{2}-7.83\times\:{\text{X}}_{3}^{2}-\:8.93\times\:{\text{X}}_{4}^{2}\end{array}$$

The findings from the regression analysis reveal that the linear effects of variables A, B, C, and D, alongside the quadratic effect of X_i_^2^, and the linear interaction influence between independent parameters are statistically significant (p-value < 0.05). The positive coefficients associated with the linear and quadratic components indicate a favourable and amplifying impact on the performance of the process, while coefficients with a negative value (-) are indicative of a detrimental effect on the process yield. Furthermore, the cooperative and conflicting influences are represented by positive and negative coefficients associated with the interaction terms, respectively.

**Table 3:** The ANOVA results for TC degradation by 25FeS/MX-450 nanocomposite.

Source	Sum of square	df	Mean square	F-value	*p*-value	Status
**Model**	7619.54	14	544.25	93.25	< 0.0001	significant
A-TC Concentration	85.06	1	85.06	14.57	0.0017	
B-Catalyst Dosage	254.63	1	254.63	43.63	< 0.0001	
C-Irradiation Time	147.68	1	147.68	25.30	0.0001	
D-pH	176.25	1	176.25	30.20	< 0.0001	
AB	2.05	1	2.05	0.3510	0.5624	
AC	283.26	1	283.26	48.53	< 0.0001	
AD	21.13	1	21.13	3.62	0.0764	
BC	111.47	1	111.47	19.10	0.0005	
BD	1287.77	1	1287.77	220.64	< 0.0001	
CD	851.15	1	851.15	145.83	< 0.0001	
A²	1904.28	1	1904.28	326.26	< 0.0001	
B²	665.90	1	665.90	114.09	< 0.0001	
C²	1682.56	1	1682.56	288.28	< 0.0001	
D²	1886.20	1	1886.20	323.17	< 0.0001	
**Residual**	87.55	15	5.84			
Lack of Fit	74.05	10	7.40	2.74	0.1385	not significant
Pure Error	13.50	5	2.70			
**Cor Total**	7707.09	29				

#### Effect of independent factors

**Fig. 5:**
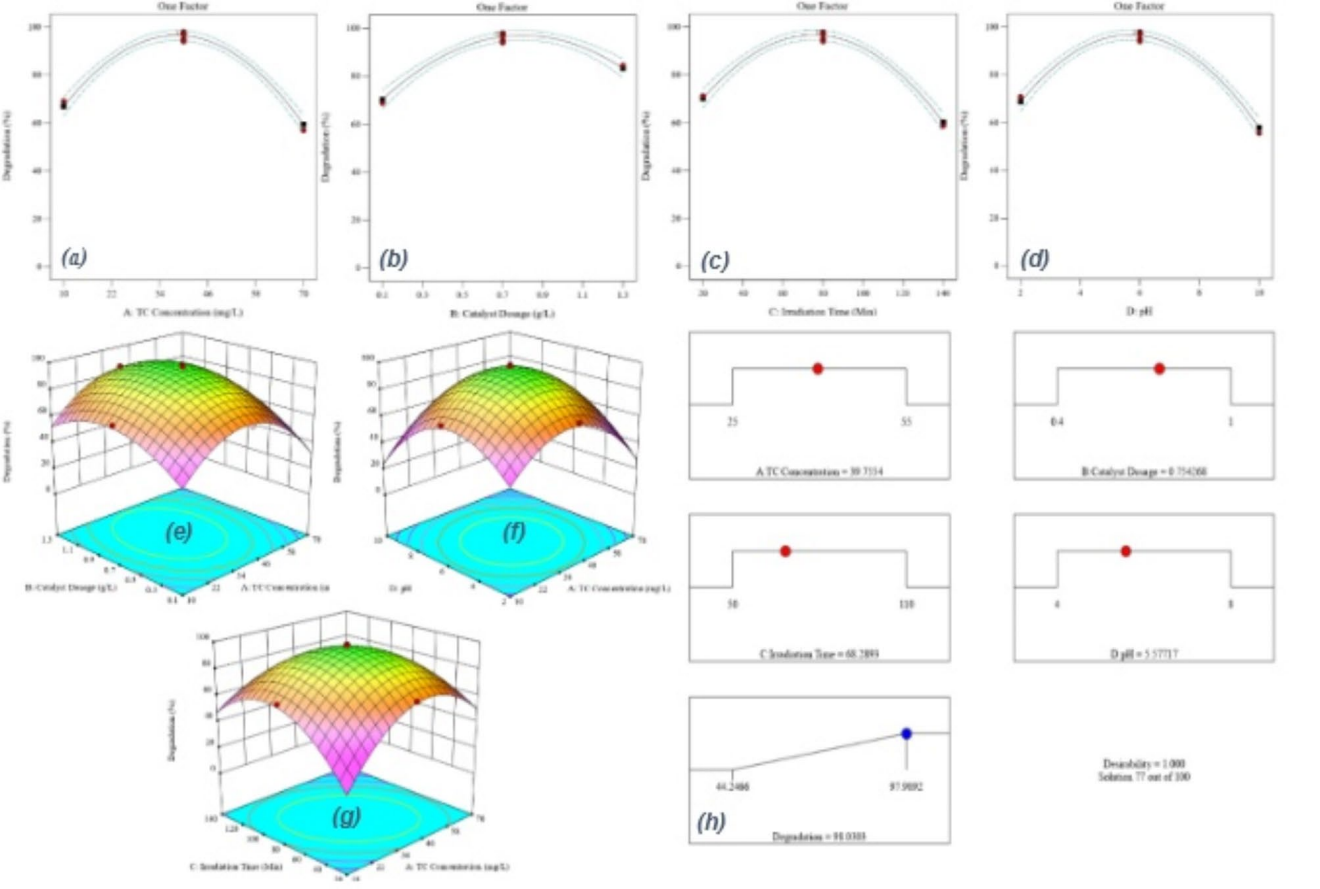
(**a**, **b**, **c**, **d**) the effect of one factor, (**e**, **f**, **g**) 3D plots of the parameters, (**h**) optimum condition of operational factors in TC degradation.

Figure 5 illustrates the impact of processing variables on degradation effectiveness. Figure 5a-d show the assessment of TC concentration and 25FeS/MX-450 nanostructure concentration on the response. The decrease in degradation efficiency with increasing pollutant concentration can be attributed to the occupation of active sites on the photocatalyst surface by the TC pollutant. Additionally, the reduction in photon availability and active site generation due to the absorption of light photons by the contaminant can justify the second reason. On the other hand, the degradation efficiency shows an increasing trend, rising from 68.7 to 97.98% as the dosage of the catalyst is raised to 0.7 g/L. This effect can be attributed to the positive impact of higher FeS dosage on the photocatalytic efficiency, stemming from the increased generation of hydroxyl radicals and superoxide radical anions due to the greater number of active sites. However, there is only a negligible improvement in degradation efficiency when the catalyst dosage exceeds 0.75 g/L (the optimal concentration). This lack of enhancement could be attributed to the reduction of active sites and low surface area for photon absorption resulting from the nanoparticle agglomeration and the decrease in the light penetration rate^15,48^.

The photocatalytic activity is significantly affected by the increase in irradiation time, as illustrated in Fig. 5d-e. The results demonstrate that 97% degradation can be attained with 80 min of visible light exposure using 0.7 g/L 25FeS/MX-450 nanoparticles. This improved performance is attributed to the higher generation of radicals resulting from the prolonged interaction between the catalyst and the pollutant^1,50^

The pH level is a critical factor in photocatalytic reactions since it directly affects degradation efficiency. The impact of different pH levels on the efficiency of TC degradation can be seen in Fig. 5f-g. TC has known pKa values of 3.3, 7.7, and 9.7, and exists in cationic (TC^+^ at pH < 3.3), zwitterionic (TC at 3.3 < pH < 7.7), and anionic (TC- at pH > 7.7) forms. The zeta potential analysis was carried out to examine the electrostatic interaction between FeS/MXene nanocomposite and TC molecules, with the findings are presented in Fig. 5f-g. The point of zero charge (PZC) is around pH = 6.2, indicating that the surface of the nanocomposite is positively charged at pH < 6.2 and negatively charged at pH > 6.2. Importantly, at lower pH levels, both the catalyst and TC are positively charged, leading to repulsion and low photocatalytic activity. In an alkaline environment, the higher concentration of OH ions engage in a competition with TC molecules to occupy the available active sites, resulting in a notable decrease in removal efficiency. Therefore, the highest degradation rate is achieved at a solution pH of 5.5, attributed to the negative charges of TC (> 3.3) and positive charge of 25FeS/MX-450 composite (< 6.2). As a result, strong electrostatic interactions between TC molecules and the catalyst led to improved adsorption capacity^33,53^.

#### Optimisation of 25FeS/MX-450 photodegradation

Design Expert software (Fig. 5h). The evaluation of the optimisation outcomes, employing the quadratic model to analyse the experimental data, indicated that the complete degradation of 39.75 mg/L of TC concentration could potentially be attained through the utilisation of 0.75 g/L of 25FeS/MX-450, at a pH of 5.57, and following an irradiation duration of 68.23 min. The experimental degradation efficiency achieved under these optimal conditions was 98.03%, confirming the model’s validity due to the close proximity between the experimental and predicted.

degradation values. The Fe_2_O_3_-SiO_2_/MXene composite, as shown in Table 4, demonstrates superior TC degradation efficiency (98%) with a shorter irradiation time and moderate catalyst dose compared to other MXene-based and conventional photocatalysts. This composite’s unique synergy of Fe_2_O_3_, SiO_2_, and MXene enhances visible light absorption, ROS generation, and electron transport, making it a promising candidate for scalable pollutant degradation in wastewater treatment.

**Table 4:** The comparison of various synthesized photocatalysts for TC degradation with the current work.

Photocatalyst	Light source	TC concentration (ppm)	pH	Catalyst dose (g/L)	Irradiation time (min)	K (min^−1^)	Degradation (%)	Ref.
CeO₂/MXene-TiO₂	550 W Halogen	30	4	0.7	95	0.051	96.46	^60^
NiCo LDHs-MXenes	300 W Xenon	20	-	0.2	50	-	97.4	^54^
IBC800 (Iron-rich sludge-derived)	300 W Xenon	10	7	0.15	300	-	95.3	^23–26^
Ti_3_C_2_-SO_3_H/g-C_3_N_4_ (TiCSOHCN	35 W Xenon	10	6	0.8	120	0.0088	75.42	^58–60^
α-Fe_2_O_3_@TiO_2_@MXene	500 W Halogen	39.37	4.8	0.54	124	-	98.36	^34^
CF/ZnO/Ag_2_O	500 W Xenon	20	5.5	-	30 (60 min Dark)	0.0837	94.5	^6^
π-COF (sp2c-COF/Py-NH_2_-COF)	300 W Xenon	20	-	0.2	90	0.0846	94.8	^16^
Bi_2_Fe_4_O_9_/rGO	300 W Xenon	10	Natural	0.2	60	-	83.73	^51^
g-C_3_N_4_ membrane reactor (PMR)	300 W xenon	22.16	9.8	0.56	113.77	-	94.80	^11^
α-Fe_2_O_3_/TiO_2_	500 W Halogen	30	5.4	0.614	161	0.02316	97.50	^28^
Fe₂O₃-SiO₂/MXene	500 W halogen	39.75	5.6	0.75	68.28	0.0283	98.00	This work

### Kinetics of the 25FeS/MX-450 nanocomposite

**Fig. 6:**
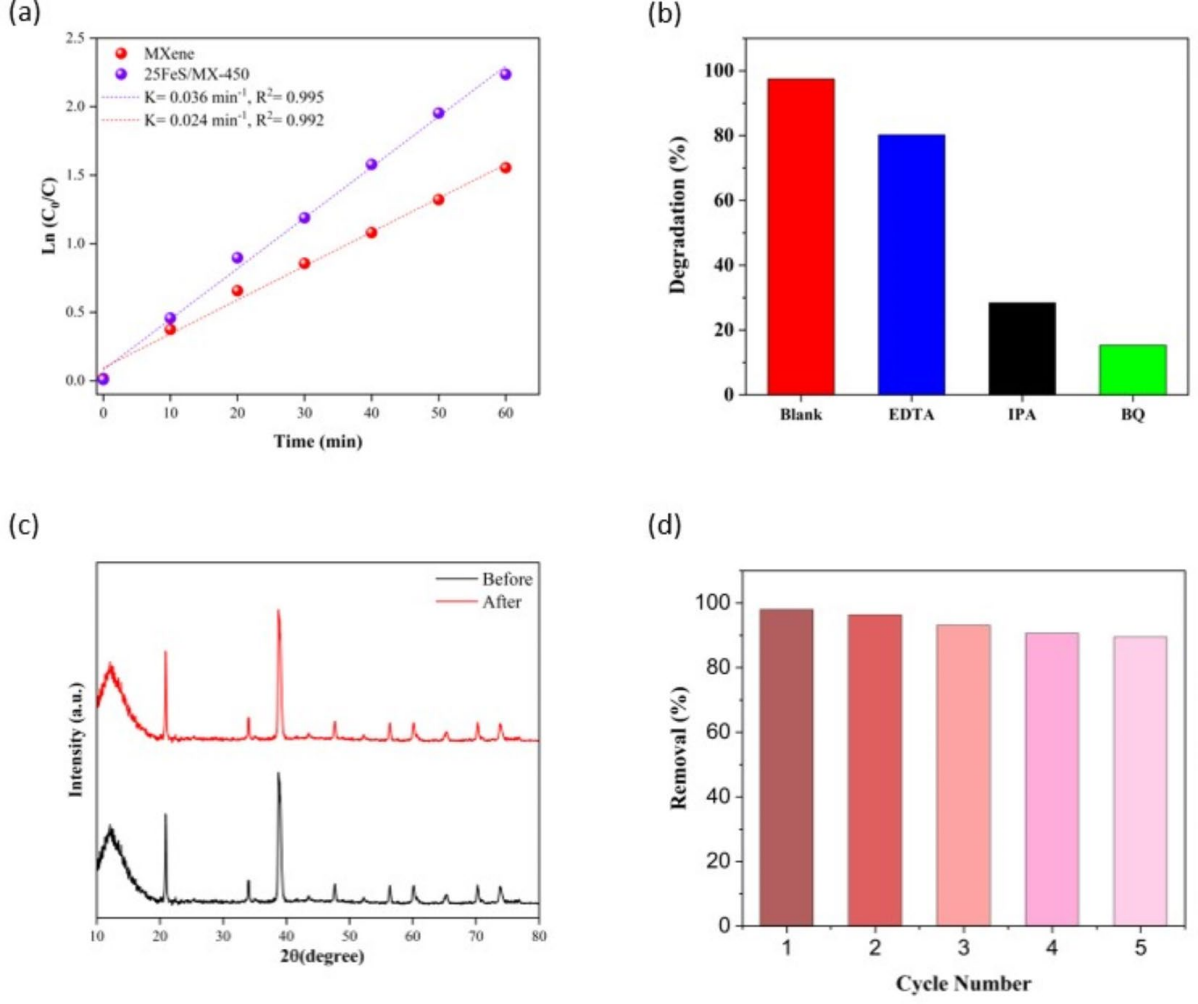
(**a**) Examination of the correlation between initial TC concentrations and first-order rate constants, (**b**) TC degradation efficiencies observed across five consecutive cycles, (**c**) X-ray diffraction (XRD) pattern of 5FeS/MX-450 before and after TC degradation, (**d**) Photocatalytic decomposition efficiency of TC utilising 25FeS/MX-450 nanocomposite with various scavengers under optimised conditions.

The decomposition of TC through photocatalysis follows the first-order kinetic model, as shown in Fig. 6a. The graph illustrates the logarithmic transformation of the ratio ln(C/C_0_) over time, wherein C_0_ and C_t_ denote the initial TC concentration and the TC concentration at a specific time “t”, respectively. The gradient of this graph corresponds to the reaction rate constant “k” which is directly associated with the degradation rate of TC^56–58^. The computational analysis results in a value of 0.0283 min^−1^ for the “k” constant when employing the 25FeS/MX-450 photocatalyst.

### Reusability and stability of 25FeS/MX-450 nanocomposite

The photocatalytic efficiency of the reused catalyst was evaluated under favourable parameters. The catalyst underwent a washing process with distilled water, repeated five times, to eliminate the adsorbed TC molecules, followed by drying and use in subsequent experimental runs. The regenerated catalyst exhibited consistent performance in breaking down the specified pharmaceutical pollutants. Subsequent to five cycles of TC breakdown, the degradation efficiency experienced a marginal decline, reaching 89.46%, as depicted in Fig. 6b. This reduction in the photocatalytic efficiency of the regenerated catalyst might be attributed to the blockage of some active sites on the catalyst’s surface by degradation by-products. However, it sustained a high level of efficacy. After 5 cycles, XRD analysis, as shown in Fig. 6c, confirmed the stability of the Fe_2_O_3_-SiO_2_/MXene composite, showing that its structure remained intact with no significant changes. This suggests that the catalyst retains its durability and performance over multiple uses, though further studies could provide additional insights into potential fouling mechanisms for even longer-term applications.

### Degradation mechanism

To thoroughly investigate the degradation mechanism of TC and obtain an insight into the specific functions of different reactive species, various scavengers of IPA, EDTA, and BQ for ^•^OH, H^+^, and ^•^O_2_^−^ radicals, respectively were used. Figure 6d shows the variation in the degradation efficiency with the introduction of various scavengers. The TC degradation rates after adding scavengers were 80.25% (EDTA), 28.35% (IPA), and 15.29% (BQ), which were significantly lower than those without scavengers. Adding benzoquinone did not remarkably affect the behaviour of the nanocomposite, indicating that the ^•^O_2_^−^ radicals produced by the photocatalyst effectively eliminate pollutants. Moreover, the presence of IPA, a scavenger of OH radicals, significantly influenced the degradation efficiency of the nanocomposite, highlighting the importance of ^•^OH^−^active species in the degradation process. However, the addition of EDTA did not have a considerable impact on the TC degradation, indicating that the presence of H^+^ does not have a substantial influence on TC degradation.

The Fe₂O₃-SiO₂/MXene composite degrades tetracycline through the generation of reactive oxygen species (ROS), facilitated by efficient electron-hole separation. Under light irradiation, photogenerated carriers produce hydroxyl radicals (•OH) and superoxide anions (•O₂⁻). The •OH radicals, formed via water oxidation, play a primary role in breaking down tetracycline, while •O₂⁻ enhances degradation through further oxidative reactions. Scavenger tests confirm that both •OH and •O₂⁻ are essential to the degradation process, highlighting the composite’s high efficiency in antibiotic removal.

**Fig. 7:**
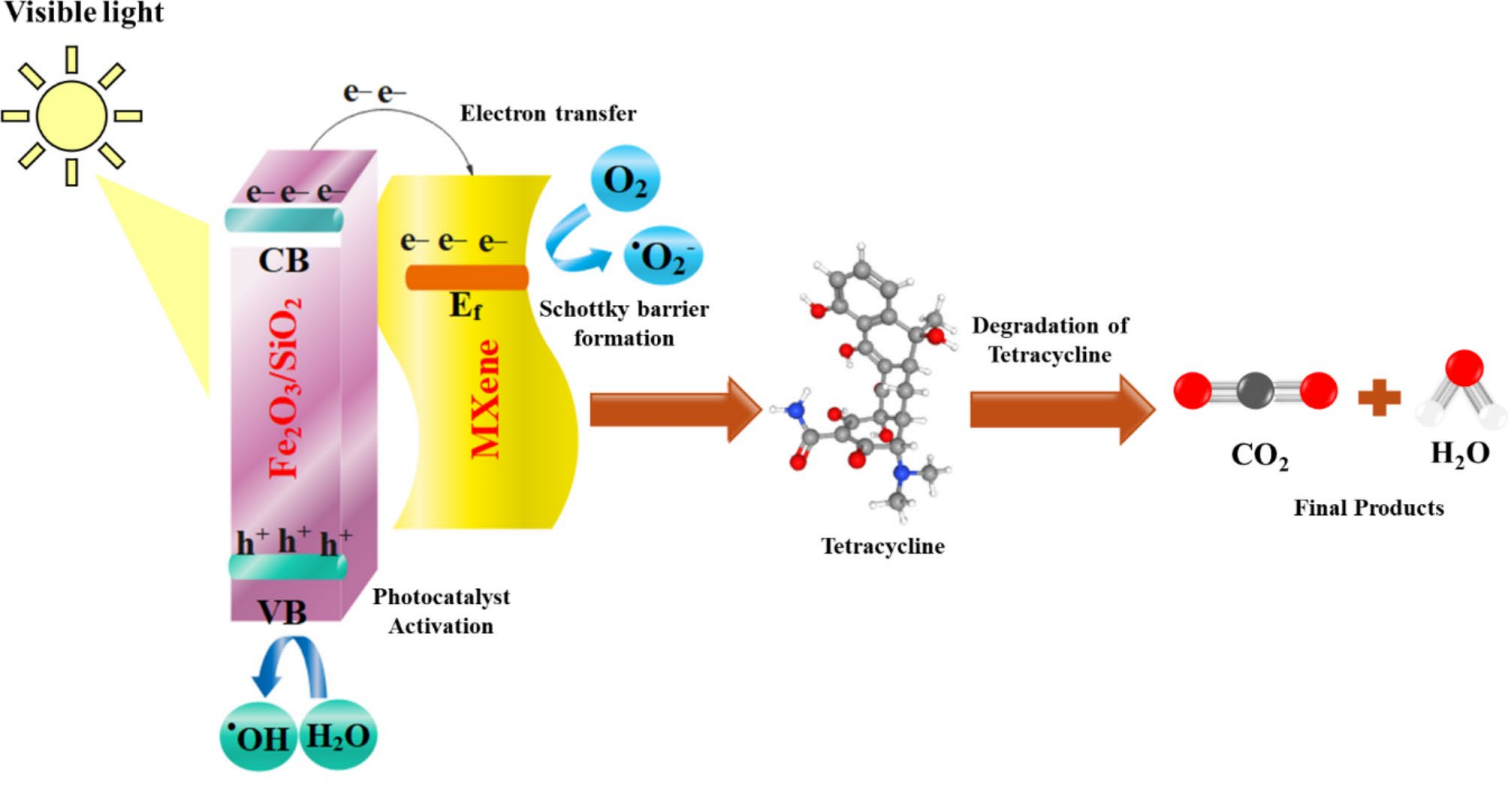
Proposed charge transfer mechanism of the 25FeS/MX-450 photocatalyst under visible light exposure.

Based on the evaluation of the photocatalyst’s performance, analysis of DRS results, and the influence of MXene on decreasing the bandgap, a proposed mechanism for the photocatalytic degradation of TC using the 25FeS/MX-450 nanocomposite is depicted in Fig. 7. The incorporation of MXene in the 25FeS/MX-450 nanocomposite facilitates the activation of FeS under visible light by reducing the bandgap and promoting efficient electron (e^−^) transfer from the valence band to the conduction band. Additionally, the formation of a Schottky barrier, attributed to the lower Fermi level of MXene compared to FeS, impedes the recombination of electron-hole pairs, thereby extending the lifetime of electrons. Moreover, photoexcited electrons migrate to the MXene surface and participate in the reaction with dissolved oxygen in water, generating ^•^O_2_^−^ radicals. Simultaneously, the photogenerated holes (h^+^) reduce H_2_O molecules adsorbed on the surface, forming ^•^OH radicals. These free radicals subsequently interact with TC molecules, leading to their degradation into non-toxic and more biodegradable byproducts. The Schottky barrier formed at the Fe₂O₃-MXene interface plays a crucial role in enabling effective charge separation within the composite. This barrier facilitates the transfer of photogenerated electrons from the conduction band of Fe_2_O_3_ to the surface of MXene, thereby minimizing electron-hole recombination a primary factor that often limits the efficiency of photocatalytic processes. This effective charge separation ensures that more electrons remain available for the generation of reactive oxygen species (ROS), such as hydroxyl and superoxide radicals. These ROS are essential for the oxidative breakdown of tetracycline, thus amplifying the overall degradation efficiency. Consequently, the Schottky barrier effect contributes directly to the enhanced photocatalytic performance of the composite by maintaining a high concentration of active charge carriers and maximizing ROS production, making the Fe_2_O_3_-SiO_2_/MXene composite a promising candidate for environmental remediation applications. In addition, inorganic ions and organic matter in real wastewater can impact the degradation efficiency of the Fe_2_O_3_-SiO_2_/MXene photocatalyst. Common ions like chloride and sulfate reduce efficiency by competing for active sites and quenching ROS, while organic matter, such as humic acid, absorbs light and competes with pollutants for ROS, thereby affecting degradation performance^13,14^.

## Conclusion

In this study, a novel Fe₂O₃/SiO₂/MXene ternary nano-photocomposite was synthesized using a rapid wet impregnation and sonochemical method, optimized specifically for tetracycline (TC) degradation in water. Characterization techniques (BET, XRD, TEM, SEM, EDX and UV-Vis) confirmed successful synthesis, showing effective incorporation of Fe₂O₃ and SiO₂ on the MXene surface. Optimal conditions for TC concentration, photocatalyst dosage, irradiation time, and pH were determined using RSM-CCD with Design Expert software, maximizing TC degradation efficiency. Under these conditions, the 25FeS/MX-450 composite achieved a high degradation rate of 98.03%, closely matching predicted values. The photocatalytic mechanism suggests that MXene’s high electron mobility supports electron transfer from FeS, generating reactive oxygen species (ROS) like superoxide (•O₂⁻) and hydroxyl radicals (•OH), which are key to TC degradation. Scavenger tests confirmed these active species as primary contributors to the process. Additionally, the composite showed strong reusability, maintaining high efficiency over five cycles, indicating practical potential in wastewater treatment. The Fe₂O₃/SiO₂/MXene composite is a promising visible-light-responsive photocatalyst for removing pharmaceutical pollutants from water. This study demonstrates its potential for sustainable water purification and highlights RSM-CCD as an effective tool for optimising photocatalytic conditions. While Fe_2_O_3_-SiO_2_/MXene composites demonstrate high efficiency in pollutant degradation, studies suggest the potential for adverse environmental impacts due to reactive oxygen species (ROS) generation, which may pose risks to aquatic life and plants. Ecotoxicological assessments are essential for evaluating these risks in real-world applications, particularly to understand the interactions of these materials within environmental systems and assess regulatory needs for safe deployment^8,10,31,36^.

The optimised Fe_2_O_3_-SiO_2_/MXene composite demonstrated exceptional photocarrier separation and light absorption capabilities, resulting in significantly higher tetracycline degradation efficiency compared to conventional photocatalysts. The ability to achieve these enhanced results is attributed to the unique combination of MXene’s rapid electron mobility and Fe_2_O_3_-SiO_2_’s photocatalytic properties, which together address common challenges in photocatalysis, such as charge recombination and limited light absorption. By employing RSM-CCD, we systematically optimized the composite’s structure and operational conditions, showcasing a scalable approach that is adaptable to various wastewater treatment applications. This study not only underscores the practical application of MXene-based composites in antibiotic removal but also sets a foundation for future research in developing high-performance, environmentally sustainable photocatalysts for complex organic pollutant degradation. The Fe₂O₃-SiO₂/MXene composite demonstrated effective photocatalytic degradation of tetracycline, with stability and reusability across multiple cycles. Future research could explore scaling up the photocatalyst for industrial applications, particularly in the pharmaceutical field, where it shows potential for the treatment of pharmaceutical contaminants in real-world wastewater conditions.

## Electronic Supplementary Material

Below is the link to the electronic supplementary material.

Supplementary Material 1

## Data Availability

The datasets employed or examined in the present study can be obtained from the corresponding authors upon reasonable request.
