# Supplementary material for: MXene-based composite photocatalysts for efficient degradation of antibiotics in wastewater
Source: Sci Rep. 2024 Dec 28;14:31498. doi: 10.1038/s41598-024-83333-3 (PMC11682083; doi:10.1038/s41598-024-83333-3)
Supplement: Supplementary file 1 — Supplementary Material 1 [file 41598_2024_83333_MOESM1_ESM.docx]

**MXene-Based Composite Photocatalysts for Efficient Degradation of Antibiotics in Wastewater**

Masoud Akbari^a^, Jamal Rasouli^c^, Kamal Rasouli^c^, Samaneh Ghaedi^d^, Milad Mohammadi^a^, Hamid Rajabi^e,^*, Samad Sabbaghi^a,b,^*

*^a^Department of Nano-Chemical Engineering, Faculty of Advanced Technologies, Shiraz University, Shiraz, Iran.*

*^b^Nanotechnology Research Institute, Shiraz University, Shiraz, Iran.*

*^c^Department of Chemical Engineering, School of Chemical and Petroleum Engineering, Shiraz University, Shiraz, Iran.*

*^d^Department of Civil Engineering and Management, the University of Manchester, Manchester M13 9PL, UK.*

*^e^Department of Civil and Environmental Engineering, School of Engineering, University of Liverpool, Liverpool L69 3GH, UK*

* Corresponding Author:

Dr. Hamid Rajabi: E-mail: [hamid.rajabi@liverpool.ac.uk](mailto:hamid.rajabi@liverpool.ac.uk)

Prof. Samad Sabbaghi: E-mail: [sabbaghi@shirazu.ac.ir](mailto:sabbaghi@shirazu.ac.ir), [samad.sabbaghi@uwaterloo.ca](mailto:samad.sabbaghi@uwaterloo.ca)

<Tel:+987136139669>, Fax: +98716139662

**Table S1**

Coded and actual levels of independent factors in CCD.

| **Factor symbol** | **Coded and levels** | | | | |
| --- | --- | --- | --- | --- | --- |
|  | -2 | -1 | 0 | 1 | 2 |
| **Calcination Temperature** | 300 | 375 | 450 | 525 | 600 |
| **Loading of Catalyst** | 5 | 15 | 25 | 35 | 45 |

**Table S2**

Arrangement of the CCD for the four independent variables and the TC removal efficiency.

| **Run No.** | **TC Concentration (mg/L)** | **Catalyst Dosage (g/L)** | **Time**  **(min)** | **pH** | **Degradation**  **(%)** |
| --- | --- | --- | --- | --- | --- |
| **1** | 40 | 0.7 | 80 | 2 | 70.7758 |
| **2** | 25 | 0.4 | 110 | 4 | 59.676 |
| **3** | 40 | 0.7 | 80 | 10 | 55.5415 |
| **4** | 55 | 1 | 50 | 8 | 59.2642 |
| **5** | 40 | 0.7 | 140 | 6 | 58.7669 |
| **6** | 25 | 1 | 50 | 8 | 51.0508 |
| **7** | 40 | 0.7 | 80 | 6 | 97.9892 |
| **8** | 40 | 0.7 | 80 | 6 | 93.9892 |
| **9** | 55 | 1 | 110 | 8 | 58.3021 |
| **10** | 40 | 0.7 | 80 | 6 | 96.9892 |
| **11** | 40 | 0.7 | 80 | 6 | 97.9892 |
| **12** | 40 | 0.7 | 80 | 6 | 94.9892 |
| **13** | 55 | 0.4 | 110 | 4 | 44.2466 |
| **14** | 25 | 0.4 | 50 | 4 | 61.64 |
| **15** | 55 | 0.4 | 50 | 8 | 68.0222 |
| **16** | 25 | 0.4 | 110 | 8 | 81.1831 |
| **17** | 25 | 1 | 50 | 4 | 91.8586 |
| **18** | 40 | 1.3 | 80 | 6 | 84.5014 |
| **19** | 10 | 0.7 | 80 | 6 | 69.07 |
| **20** | 70 | 0.7 | 80 | 6 | 56.93 |
| **21** | 55 | 1 | 50 | 4 | 97.4361 |
| **22** | 55 | 0.4 | 110 | 8 | 73.8798 |
| **23** | 40 | 0.7 | 20 | 6 | 71.2338 |
| **24** | 25 | 1 | 110 | 4 | 74.03 |
| **25** | 55 | 1 | 110 | 4 | 61.6048 |
| **26** | 25 | 0.4 | 50 | 8 | 61.2355 |
| **27** | 40 | 0.7 | 80 | 6 | 96.9892 |
| **28** | 40 | 0.1 | 80 | 6 | 68.7391 |
| **29** | 55 | 0.4 | 50 | 4 | 64.2716 |
| **30** | 25 | 1 | 110 | 8 | 67.2568 |


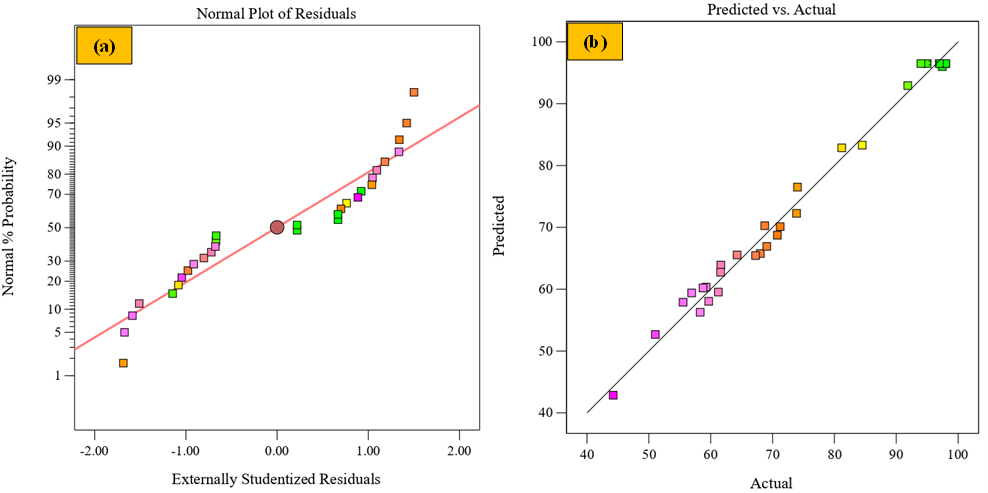


**Fig. S1.** a, b) Plots of normal probability vs. residuals and predicted vs. actual values for TC.
